# Transcriptomic analysis identifies lactoferrin-induced quiescent circuits in neonatal macrophages

**DOI:** 10.3389/fimmu.2023.1276173

**Published:** 2023-10-06

**Authors:** Michael Eigenschink, Isabelle Wessely, Marco Dijmarescu, Elisabeth Förster-Waldl, Alex Farr, Herbert Kiss, Angelika Berger, Lukas Wisgrill

**Affiliations:** ^1^ Division of Neonatology, Pediatric Intensive Care and Neuropaediatrics, Department of Pediatrics and Adolescent Medicine, Comprehensive Center for Pediatrics, Medical University of Vienna, Vienna, Austria; ^2^ Department of Obstetrics and Gynecology, Division of Obstetrics and Feto-Maternal Medicine, Comprehensive Center for Pediatrics, Medical University of Vienna, Vienna, Austria

**Keywords:** human lactoferrin, neonatal immune system, immune tolerance, macrophage function, microbiome, necrotizing enterocolitis, innate immunity, mucosal immunity

## Abstract

**Introduction:**

Upon birth, a hitherto naïve immune system is confronted with a plethora of microbial antigens due to intestinal bacterial colonization. To prevent excessive inflammation and disruption of the epithelial barrier, physiological mechanisms must promote immune-anergy within the neonatal gut. As high concentrations of human lactoferrin (hLF), a transferrin glycoprotein shown to modulate macrophage function, are frequently encountered in colostrum, its direct interaction with intestinal macrophages may satisfy this physiological need. Thus, the primary objective of this study was to investigate transcriptional changes induced by human lactoferrin in neonatal monocyte-derived macrophages.

**Methods:**

Cord blood-derived monocytes were differentiated with M-CSF in presence or absence of 500 µg/mL hLF for 7 days and afterwards stimulated with 1 ng/mL LPS or left untreated. RNA was then isolated and subjected to microarray analysis.

**Results:**

Differentiation of cord blood-derived monocytes in presence of hLF induced a distinct transcriptional program defined by cell cycle arrest in the G2/M phase, induction of IL-4/IL-13-like signaling, altered extracellular matrix interaction, and enhanced propensity for cell-cell interaction. Moreover, near-complete abrogation of transcriptional changes induced by TLR4 engagement with LPS was observed in hLF-treated samples.

**Discussion:**

The global transition towards an M2-like homeostatic phenotype and the acquisition of quiescence elegantly demonstrate the ontogenetical relevance of hLF in attenuating pro-inflammatory signaling within the developing neonatal intestine. The marked anergy towards proinflammatory stimuli such as LPS further underlines the glycoprotein’s potential therapeutic relevance.

## 1 Introduction

Human lactoferrin (hLF) is an 80 kDa, 703 amino acid-long protein belonging to the conserved family of transferrin glycoproteins (1). Structurally, it consists of two globular lobes connected by a three-turn helix, capable of binding ferric iron (Fe^3+^) with higher affinity than serum transferrin (2, 3). Displaying a low dissociation constant at acidic pH, lactoferrin is thought to act in concert with plasma-transferrin by reducing iron availability in the microenvironment of inflamed tissue. This activity aids the inhibition of bacterial RNA- and DNA-synthesis, metabolism, enzymatic function, and ultimately microbial growth, virulence and pathogenicity (4). HLF’s functional properties are also exploited by human immune cells, as the molecule is highly abundant in secondary and tertiary granules of granulocytes. Vastly mobilized during the initial stages of host-pathogen interaction, 10^6^ granulocytes may release up to 15 µg of hLF (5). Therefore, elevated local concentrations of hLF are present in inflamed tissue and shape the microenvironment at the site of immunological action (6).

In addition to its bacteriostatic and tissue-protective function, hLF also elicits direct bactericidal effects (7), and interacts with the cellular fraction of the immune system (8). The latter is especially important to the function of human monocytes. These cells enter the site of infection and differentiate into macrophages in an hLF-rich microenvironment. As their role largely depends on polarization into M1- or M2-phenotypes, it is tempting to speculate that direct interactions of hLF with monocyte lactoferrin receptor (MC-LfR) and low-density lipoprotein receptor-related proteins (LRPs) shape distinct cellular responses (9, 10).

Adding an additional layer of complexity, both local and systemic lactoferrin concentrations follow a distinct trajectory over time. In term-born infants, lactoferrin reaches systemic levels between 200 and 800 µg/L within the first two weeks of life (11). Considering its high concentration in colostrum (up to 7 g/L), hLF seems pivotal for the development of a favorable neonatal gut-microbial composition and maintenance of intestinal barrier integrity in newborns (12).

Although macrophages have been shown to be crucially involved in maintaining intestinal barrier function (13) and hLF appears to prevent the development of intestinal hyperinflammation in preterm infants (14) by inducing a distinct anti-inflammatory/anergic state (15), a deeper understanding of the transcriptional networks that regulate the activity of neonatal macrophages upon birth is currently missing. Therefore, we aimed to identify the transcriptional profile of hLF-treated neonatal monocyte-derived macrophages and the mechanisms that underlie its immunomodulatory properties. We were able to show that hLF drastically alters the cell cycle progression of neonatal macrophages, leading to the development of a distinct transcriptional program that drives polarization towards an M2-tissue homeostatic phenotype unresponsive to pro-inflammatory stimuli.

## 2 Materials and methods

### 2.1 Study population

The umbilical cord blood of term-born infants (n = 4, 38-40 weeks of gestation) was collected aseptically into heparin tubes after cesarean section. Infants with congenital malformations and congenital infections and infants whose mothers suffered from autoimmune disease, or inborn or acquired immune deficiencies were not considered eligible for study inclusion. All procedures were approved by the ethics committee of the Medical University of Vienna (ref. 1923/2012) and informed consent was obtained from the mothers.

### 2.2 Cell isolation and culture

Ficoll-Paque density gradient centrifugation was used to enrich peripheral blood mononuclear cells (PBMCs). CD14^+^ monocytes were subsequently isolated from the PBMC-fraction using magnetic bead separation (anti-CD14 MicroBeads, Miltenyi Biotec, Bergisch-Gladbach, Germany). Consequently, 0.75 × 10^6^ cells/mL were transferred into a 6-well flat-bottomed plate (Greiner Bio-One, Kremsmünster, Austria), cultured in RPMI-1640 medium supplemented with 10% fetal calf serum (FCS) (GIBCO, Carlsbad, CA, USA) and differentiated using macrophage colony-stimulating factor (M-CSF) (100 ng/ml; PeproTech, Rocky Hill, NJ, USA) in the absence or presence of hLF (500 µg/ml; Sigma Aldrich, St Louis, MO, USA). Cells were kept in an incubator for 7 days at 37°C, 5% CO_2_, and 95% humidity and either stimulated with ultrapure lipopolysaccharide (LPS, 1 ng/mL, E. coli O111:B4, InvivoGen, San Diego, CA, USA) 24h prior to RNA-extraction or left unstimulated.

### 2.3 RNA-extraction and microarray processing

Total RNA was isolated from 1.5 x 10^6^ differentiated macrophages using the RNeasy Plus Micro Kit (Qiagen, Venlo, the Netherlands) according to the manufacturer’s instructions. RNA was then quantified using a NanoDrop 8000 device (Thermo Scientific, Waltham, MA, USA) and RNA integrity number (RIN) was determined employing the RNA 6000 Nano Kit (Agilent Technologies, Santa Clara, CA, USA). To prepare samples for microarray analysis, fragmented and biotin-labeled sense-stranded cDNA was prepared from 100 ng of total RNA (20 ng/µl) using the GeneChip Whole Transcript (WT) PLUS Reagent Kit (Affymetrix, Santa Clara, CA, USA). cDNA was then hybridized to the GeneChip Human Gene 2.1 ST Array Plate (Affymetrix, Santa Clara, CA, USA) and automatically processed and analyzed using the GeneTitan instrument (Affymetrix, Santa Clara, CA, USA).

### 2.4 Bioinformatic analyses

RNA-microarray data were processed employing the *maEndToEnd Affymetrix differential gene expression workflow* (16). Briefly, data were background-corrected, normalized, and summarized using the Robust Multi-array Average (RMA) algorithm with quantile normalization before intensity-based filtering. Afterwards, transcript clusters were annotated using the *hugene21sttranscriptcluster.db* package (17) and differential gene expression analyzed using *limma* (18). Visualizations were performed employing the R packages *ggplot2* (19)*, VennDiagram* (20), *networkD3* (21), *GoPlot* (22), and *ComplexHeatmap* (23).

#### 2.4.1 Gene set enrichment analysis

For gene set enrichment analysis (GSEA), genes with similar expression values to respective differentially expressed genes (DEG) were selected from the dataset and used as a background for each experimental condition (16). *Reactome Pathway Enrichment Analysis* was then carried out using *ReactomePA* (24), biological theme comparison performed with *clusterProfiler* (25), and results depicted utilizing the *enrichplot* (26) package. Chord diagrams were generated with *GoPlot* (22) by manually computing respective adjacency matrices between DEG and enriched pathways. Results were filtered for biologically meaningful interactions to reduce noise, by only selecting genes with a logFC higher than 2.5 or lower than -2.5 for visualization.

#### 2.4.2 Gene regulator enrichment analysis

For Gene Regulator Enrichment Analysis, normalized expression values were used as input for the *RegEnrich* algorithm (27). Thus, using a predefined set of human transcription regulators, a regulator-target network inference based on weighted gene co-expression network analysis was conducted. Subsequently, enrichment analysis was performed using Fisher’s exact test and the RegEnrich score, a metric corresponding to the biological importance of respective regulators, was modeled. Data were visualized in a heatmap by extracting the normalized expression values of the top-25 regulators for each respective condition. Additionally, RegEnrich scores of the top-20 regulatory molecules were depicted as a bar chart. Using the previously computed adjacency matrices of enriched pathways and differentially expressed genes, a second matrix that numerically estimates the effect each transcriptional regulator exerts on a cellular function was created. This was achieved by assigning a point to a regulator for each shared gene with a respective pathway and extracting the five regulators with the highest scores. Thus, functional relations between transcription factors and enriched pathways were modeled and visualized in a Sankey plot using the *networkD3* package (21). Furthermore, to depict connectivity between transcriptional regulation and biological functionality, a protein-protein interaction network (PPI) was generated using the *STRING database* (28) (Version 11.5) for proteins shown to be involved in the regulation of enriched pathways upon hLF-treatment and all DEG found in the dataset. The retrieved network was then filtered for entries with a minimum required interaction score of 0.900 (highest confidence), exported, and processed in *Cytoscape* (29) (Version 3.10.0).

#### 2.4.3 Cytokine expression and membrane protein abundance

To analyze differences in cytokine expression between conditions, DEG were filtered for cytokines annotated in the *ImmPort Cytokine Registry* (30). Similarly, using *InnateDB’s* (31) annotation of proteins relevant to innate immunity and the *Human Protein Atlas’* (32) annotation of subcellular protein locations, DEG relevant to the innate surfaceome of macrophages were extracted. Additionally, a more targeted analysis of immunologically relevant genes was conducted. Differences between target means were calculated using ANOVA and Tukey’s *post-hoc* test employed to retrieve adjusted p-values for pairwise comparisons.

#### 2.4.4 Cell-cell interaction model

With the purpose of modelling the propensity of hLF-treated macrophages to interact with other immune cells via direct physical- or indirect soluble-interactions, all DEG of membrane proteins and cytokines were extracted from the dataset using annotations stored in the *ImmPort Cytokine Registry* (30) and *the Human Protein Atlas* (32). Moreover, experimentally curated physical protein-protein interactions annotated in *InnateDB* (31), as well as cytokine-receptor interactions denoted in the *KEGG pathway database* (33), were retrieved. Subsequently, two adjacency matrices incorporating normalized expression data of selected immune cells stored in the *RNA Monaco immune cell gene data* (34) available from *The Human Protein Atlas* (32) and our experimental results, were modeled. Because the product of two interacting proteins within adjacency matrices of standardized expression sets yields a constant arbitrary value, the sum of respective products per interacting cell-pair produces a metric for interaction likelihood between two cells. Thus, differences in interaction probability between treated macrophages and all immune cells present in the *RNA Monaco immune cell gene data* (34) were determined and depicted in a radarchart using the R package *fmsb* (35). Additionally, mean expression of cytokines and membrane proteins with the highest difference in interaction scores between uMϕ+LPS, hlfMϕ +LPS were retrieved, ordered according to their number of interactions with other cells (“interaction score”), and displayed as a bar chart.

#### 2.4.5 Statistical analyses

For differential gene expression analysis, we employed the empirical Bayes variance moderation method to compute moderated t-statistics. If not indicated otherwise within this section, we selected genes with a false discovery rate (FDR) < 0.05 and log2-fold changes below -0.58 and above 0.58, respectively, for subsequent analyses. Pathway enrichment analysis was performed on input genes with an FDR < 0.1 in line with the statistical settings recommended in the *maEndToEnd Affymetrix differential gene expression workflow* (16). For biological theme comparison, DEG with an FDR < 0.05 and log2-fold changes below -0.58 or above 0.58 were used as input. Transcription regulator network target inference with RegEnrich (27) was performed by constructing an undirected, unsigned co-expression network on differentially expressed genes employing a minimum scale-free topology fitting index (R^2^) of 0.85 and retaining the top 5% of edges in the full network. Regulator enrichment was subsequently calculated for genes with a differential analysis p-value < 0.05, using a cutoff of 0.05 for adjusted enrichment p-value and 0.2 for enrichment q-value.

## 3 Results

### 3.1 HLF induces a distinct transcriptional pattern capable of repressing LPS-induced effects

**Figure 1:**
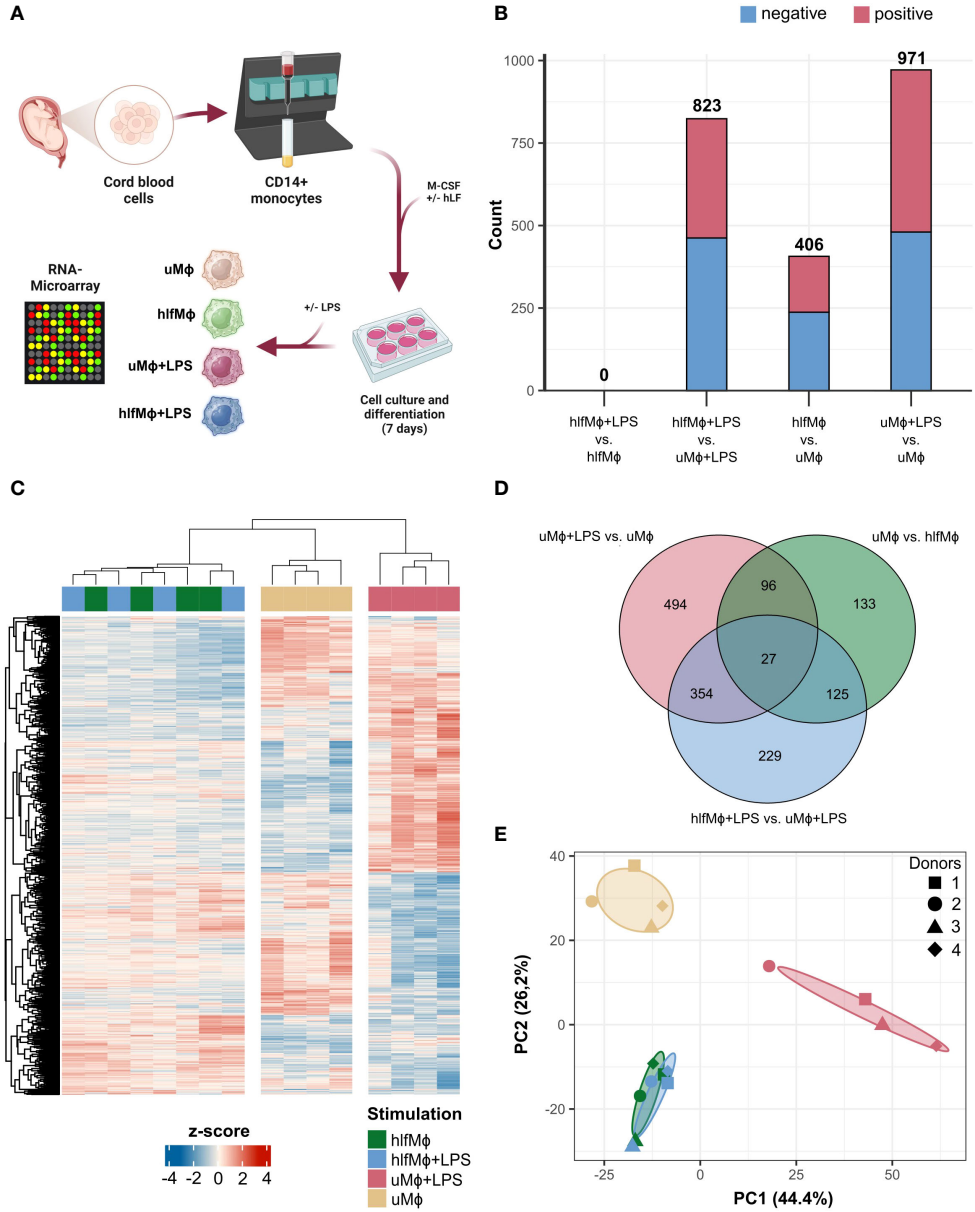
Neonatal macrophages differentiated in the presence of hLF reveal a distinct RNA-signature. **(A)** Cord-blood derived neonatal monocytes were isolated and differentiated into macrophages in the presence or absence of hLF for seven days and stimulated with LPS. RNA was isolated and analyzed using microarray technology (Created with BioRender.com). **(B)** Bar plot depicting the count of negatively (blue) and positively (red) differentially expressed genes (DEG) between experimental conditions. **(C)** DEG of all conditions hierarchically clustered using Euclidean distance as a similarity measure, displayed as a heatmap. **(D)** Venn Diagram of unique and shared DEG between respective treatments. **(E)** Principal component analysis of macrophage expression profiles.

Highly abundant in breast milk, human lactoferrin exerts a diverse set of immunological and developmental functions. Next to direct bactericidal effects, hLF might also act as an immunological primer of macrophages, a cell subset capable of propagating inflammatory damage in the evolving human gut. Thus, we established an *in vitro* culture system to differentiate neonatal monocytes into macrophages in the presence or absence of hLF. Additionally, we used LPS-treatment to mimic pro-inflammatory stimuli encountered in the intestine upon bacterial colonialization (**Figure 1A**). Thus, following our previous work on hLF-mediated attenuation of pro-inflammatory macrophage polarization, we analyzed transcriptomic changes elicited by hLF to decipher biological patterns that underlie its manifold of immunological functions (15). Transcriptomic signatures of macrophages differentiated with M-CSF in the absence (uMϕ) or presence (hlfMϕ) of hLF, either stimulated with LPS (uMϕ+LPS, hlfMϕ+LPS) or left untreated (uMϕ, hlfMϕ), were investigated using RNA microarray technology. Differential gene expression analysis revealed 406 differentially expressed genes (DEG) between hlfMϕ and uMϕ. Moreover, stimulation of uMϕ with LPS induced an increase in transcriptional activity (971 DEGs) that vastly differed from that observed in hlfMϕ+LPS (823 DEGs). This was of particular interest, as the comparison between hlfMϕ and hlfMϕ+LPS revealed no DEG (**Figure 1B**). To completely exclude the existence of DEG between hlfMϕ and hlfMϕ+LPS we re-analyzed the comparison without employing logFC cut-offs. Even after this adaptation, no DEG were found between the two conditions (**Supplementary Figure 1**). Correspondingly, hierarchical clustering of DEG deriving from all conditions revealed a distinct transcriptomic profile of hLF-treated cells when compared to uMϕ and uMϕ+LPS (**Figure 1C**). This finding is also in line with the high expressional overlap between stimulated and unstimulated hlfMϕ observable in the Venn diagram (**Figure 1D**) and the clear separation between uMϕ, uMϕ+LPS and hLF-treated cells shown by principal component analysis (**Figure 1E**).

Taken together, the differentiation of neonatal monocytes in the presence of hLF induced a distinct transcriptomic profile and severely impaired the responsiveness of derived macrophages to LPS stimulation.

### 3.2 Human lactoferrin modulates cell cycle progression and induces M2 polarization

**Figure 2:**
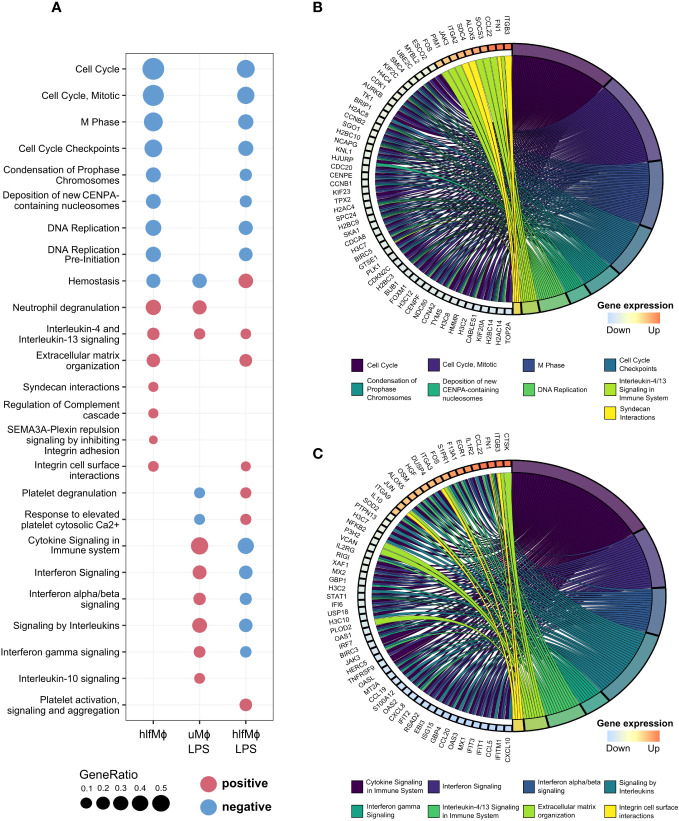
Functional enrichment analysis unravels cell cycle regulation as a driver of hLF-mediated quiescence. Functional Enrichment Analysis was performed using ClusterProfiler. **(A)** Biological theme comparison between treatment conditions depicted as a dot plot. Upregulated pathways are highlighted in red, downregulated pathways marked blue. **(B, C)** Chord diagrams showing the involvement of genes with logFC-values higher than 2.5 and lower than -2.5 in pathways discovered during functional enrichment analysis. Graphs correspond to differential gene expression matrices hlfMϕ vs. uMϕ **(B)** and hlfMϕ+LPS vs. uMϕ+LPS **(C)**, respectively.

To understand the cellular responses hLF evokes in neonatal monocyte-derived macrophages, a gene set enrichment analysis (GSEA) was performed using the *Reactome* database of curated cellular pathways (**Figure 2A**). Biological theme comparison revealed cell-cycle specific effects of hLF, as displayed by marked downregulation of cell cycle progression, DNA replication, and deposition of CENP-A nucleosomes needed for maintaining centromere identity. Moreover, hLF-treated cells presented with an abrogation of LPS-induced effects on cytokine signaling, including engagement in interferon-alpha/beta and interferon-gamma pathways, as exemplified by diametrically opposed enrichment results, whilst concomitantly maintaining cell-cycle specific properties (**Figure 2A**). To gain a deeper understanding of the drivers of hLF-function and its immunosuppressive properties, chord diagrams displaying the most disparate expression profiles for respective *Reactome* pathways were generated (**Figures 2B, C**). In this context, genes of several histones, proteins (*NDC80, CENPF, SKA1, TPX2*), cyclins (*CCNA2, CCNB1, CCNB2*), and cyclin-dependent kinases (*CDK1*) were downregulated in hlfMϕ when compared to uMϕ - all of which relevant to chromosome segregation, kinetochore function, or cell cycle regulation. In addition, hLF seemed to promote interactions with extracellular matrix proteins via upregulation of transmembrane receptors *ITGB3* and *ITGA2* as well as *FN1* and *SCD4*. It also triggered downstream effects comparable with IL-4- and IL-13-induced signaling by increasing the expression of *JAK3, FOS, SOCS3, ALOX5, CCL22*, and *PIM1* (**Figure 2B**). Additionally, the upregulation of anti-inflammatory signaling molecules *FOS, EGR1*, and *JUN*, as well as increased expression of decoy receptor *IL1R2* and anti-inflammatory cytokine *IL-10* seem to stand in stark contrast to an LPS-induced proinflammatory phenotype (**Figure 2C**). In line with this finding, hLF also potently blocked LPS-induced upregulation of cytokines and chemokines *CXCL8, CXCL10, CCL5, EBI3*, and *CCL20* as well as interferon-stimulated genes *IFITM1, ISG15, IFIT1*, and *OAS2*.

Overall, functional analyses revealed that hLF-induces a ‘quiescent’ state in macrophages due to modification of cell cycle regulation, an important mechanism recently described to drive macrophages into an M2-like phenotype (36).

### 3.3 HLF induces distinct gene regulatory networks that drive neonatal macrophages into quiescence

**Figure 3:**
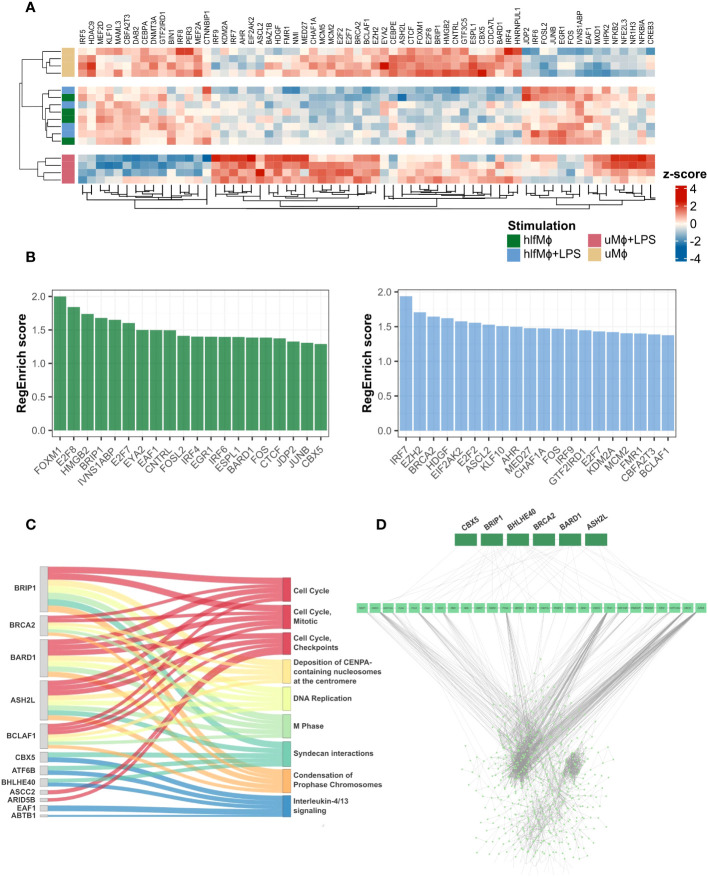
Gene regulator enrichment analysis identifies key-regulators of hLF-function and reveals their high transcriptomic connectivity. Transcription regulator-target network inference based on weighted gene co-expression analysis was performed using RegEnrich. **(A)** Heatmap depicting mean expression values of the top-25 transcription regulators per condition, employing Euclidian distance as a similarity measure. **(B)** Bar chart showing the 20 regulators with the highest RegEnrich score for hlfMϕ (green) and hlfMϕ+LPS (blue), respectively. **(C)** Using the adjacency matrix previously computed to display the influence of gene expression on enriched Reactome pathways, the top-five genetic regulators for each enriched pathway were identified in hlfMϕ and their contribution to biological function visualized using a Sankey plot. **(D)** Regulators involved in the control of hLF-induced biological functions were retrieved and a protein-protein interaction network constructed that displays their contribution to the regulation of all DEG found in the dataset.

In order to understand the gene regulatory programs that underlie hLF-induced quiescence, we performed a gene regulator enrichment analysis using *RegEnrich* (27). This algorithm identifies genetic regulators based on differential gene expression analysis and regulator-target network inference, and provides a metric for their regulatory importance. Hierarchical clustering of the top-25 regulators per condition revealed prominent changes in the mean expression of gene regulatory molecules in hLF-treated samples, irrespective of LPS stimulation (**Figure 3A**). These gene regulatory changes were driven by the downregulation of cell cycle regulatory proteins *FOXM1*, *E2F8*, *HMGB2*, and *BRIP1* and the upregulation of immunomodulatory protein *IVNS1ABP*. Additionally, downregulation of pro-inflammatory regulators *IRF7*, *EZH2*, *HDGF*, and *EIF2AK2* was observed when comparing hlfMϕ+LPS to uMϕ+LPS, highlighting that hLF induces transcriptional unresponsiveness towards pro-inflammatory LPS-stimuli (**Figure 3B**). To better understand the functional implications of hLF-mediated transcriptional regulation, the top-five regulators per pathway found to be enriched in hlfMϕ were retrieved. This analysis revealed the importance of *BRIP1*, *BRCA2*, *BARD1*, *ASH2L*, and *BCLAF1* as functional regulators of cell-cycle progression and *CBX5*, *ATF6B*, *BHLHE40*, *EAF1*, and *ABTB1* as mediators of IL-4/IL-13-like effects (**Figure 3C**). Because RegEnrich – per default – infers a regulatory network on all significantly expressed genes, irrespective of their logarithmic fold change value (logFC), a protein-protein interaction network (PPI) for genetic regulators of hLF-induced functions and differentially expressed genes that displayed a log2-fold change of 0.57 or higher was constructed. Although functional regulators interacted with only 27 proteins directly, network analysis revealed high connectivity with all differentially expressed genes identified in our experimental model (**Figure 3D**).

In concert, these results highlight the tight connection between cell-cycle regulators and functional heterogeneity in hlfMϕ and underline their importance in the development of a quiescent state that remains transcriptionally irresponsive to immunogenic stimuli.

### 3.4 HLF-induced quiescent macrophages display a tissue-homeostatic interactome

**Figure 4:**
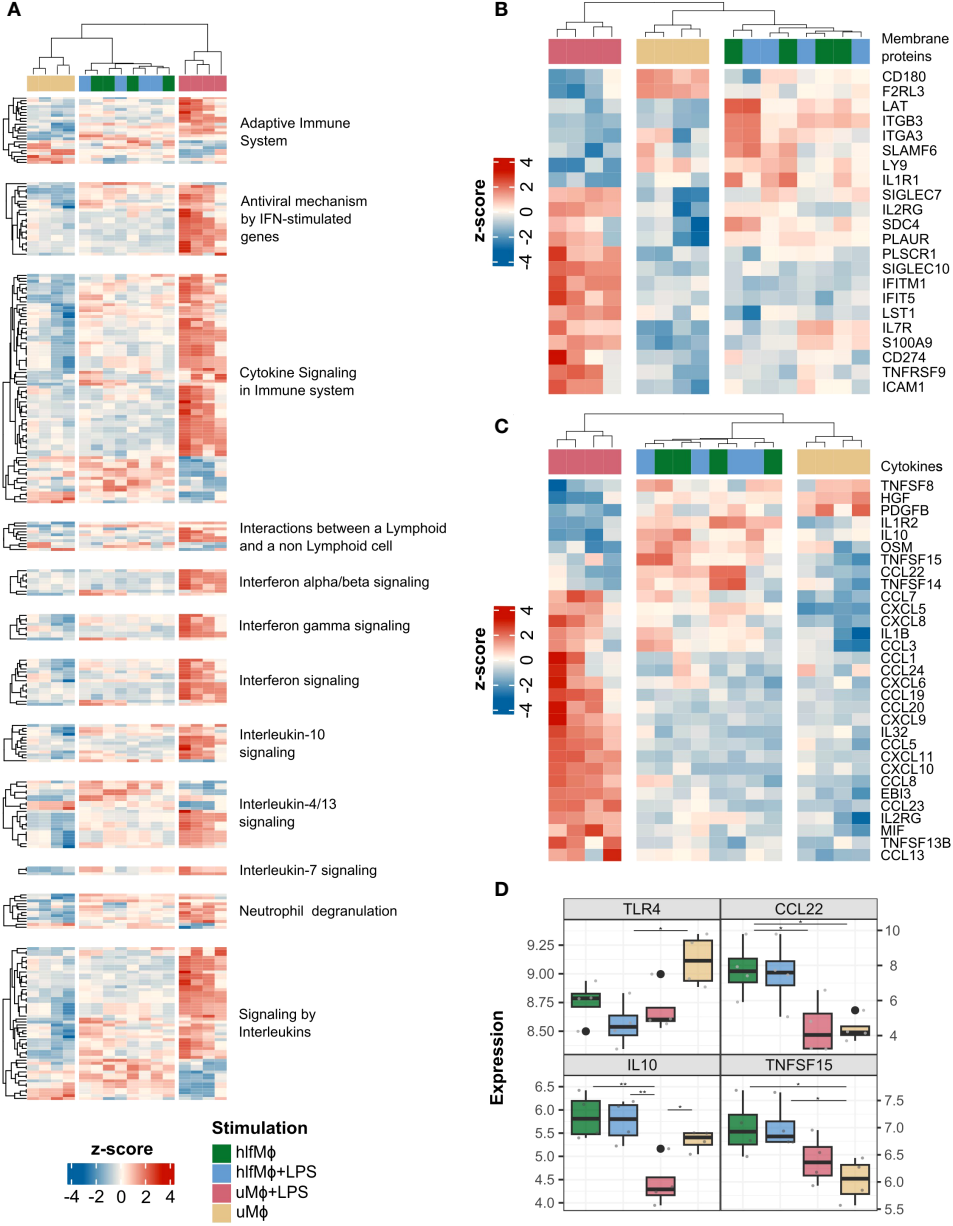
HLF-treatment induces a homeostatic interactome in neonatal macrophages. Using the annotated databases ImmPort, Human Protein Atlas and ImmuneDB, DEG of cytokines and membrane proteins were retrieved from the dataset. **(A)** A heatmap depicting DEG of cytokines and immunologically important membrane proteins was generated using Euclidian distance as a similarity measure for immunologically relevant enriched pathways of all experimental conditions. A similar visualization was performed for all membrane protein- **(B)** and cytokine- **(C)** related DEG found in the dataset, irrespective of enriched pathways. **(D)** Gene expression values of TLR4, CCL22, IL-10 and TNFSF15 were plotted as boxplots and significance indicated with lines and asterisks for p < 0.05 (*), and p < 0.01 (**), respectively.

As our initial analyses pointed towards induction of a quiescent M2-like state, we aimed to explore whether this homeostatic phenotype also extends to the interactome of hLF-treated macrophages. Harnessing *ImmuneDBs* data on immunologically relevant genes, *Immports* cytokine registry data, and information on subcellular protein localization available from *Human Protein Atlas*, membrane protein, and cytokine gene abundances were computed for each experimental condition. Global changes in cytokine and membrane protein gene expressions were evident for all immunologically implicated enriched *Reactome* pathways (**Figure 4A**). Upon closer examination, hlfMϕ displayed modified expression of *PLAUR*, *ICAM1*, *ITGB3*, and *ITGA3*, arguing for changes in cell adhesion and matrix interaction properties. Moreover, together with the altered presence of *CD180*, *Ly9*, *LAT*, and *PLSCR1*, these changes suggested modifications in phagocytotic and efferocytotic functions. Complementing this homeostatic interactome are the increased expression of *SIGLEC7*, *SDC4*, *S100A9*, and *TNFRSF9*, proteins that have been ascribed anti-inflammatory properties (**Figure 4B**). Regarding soluble interactions, upregulation of homeostatic and gut protective cytokines *IL-10*, *TNFSF14*, and *CXCL8* was seen in macrophages treated with hLF. Moreover, hlfMϕ displayed an increased expression of cytokines involved in regulating the proliferation and activity of mucosal innate lymphoid cells. Contradictory at first glance, hLF also mediated the expression of pro-inflammatory molecules *OSM*, *CCL7*, *CXCL5*, *IL1B*, and *CCL3*. Yet globally, hlfMϕ+LPS displayed substantial decreases in the expression of pro-inflammatory molecules when compared to uMϕ+LPS (**Figure 4C**). To additionally validate our findings, we employed ANOVA with *post-hoc* Tukey-correction to exclude a solely TLR4 dependent reduction of LPS-signaling in hlfMϕ and statistically support the increases observed in IL-10, CCL22 and TNFSF15 production – cytokines detrimental to intestinal homeostasis (**Figure 4D**).

Collectively, these results argue for the induction of a quiescent, tissue-homeostatic interactome in hLF-treated neonatal macrophages. Moreover, the near complete abrogation of LPS-induced effects suggests that hLF efficiently drives the cellular communication of neonatal macrophages towards tolerance.

### 3.5 HLF increases physical cell-cell interaction propensity and attenuates LPS-induced cytokine crosstalk

Due to the marked changes hlfMϕ displayed in membrane protein and cytokine expression, we next evaluated their global propensity to interact with other immune cells. Thus, we combined the previously computed membrane protein and cytokine expression data with *Innate DB’s* manually curated annotation of immunologically relevant physical protein-protein interactions, cytokine-receptor interactions stored in the *KEGG pathway database*, and normalized gene expression values available from the *Monaco immune cell gene data* provided by the *Human Protein Atlas*.

**Figure 5:**
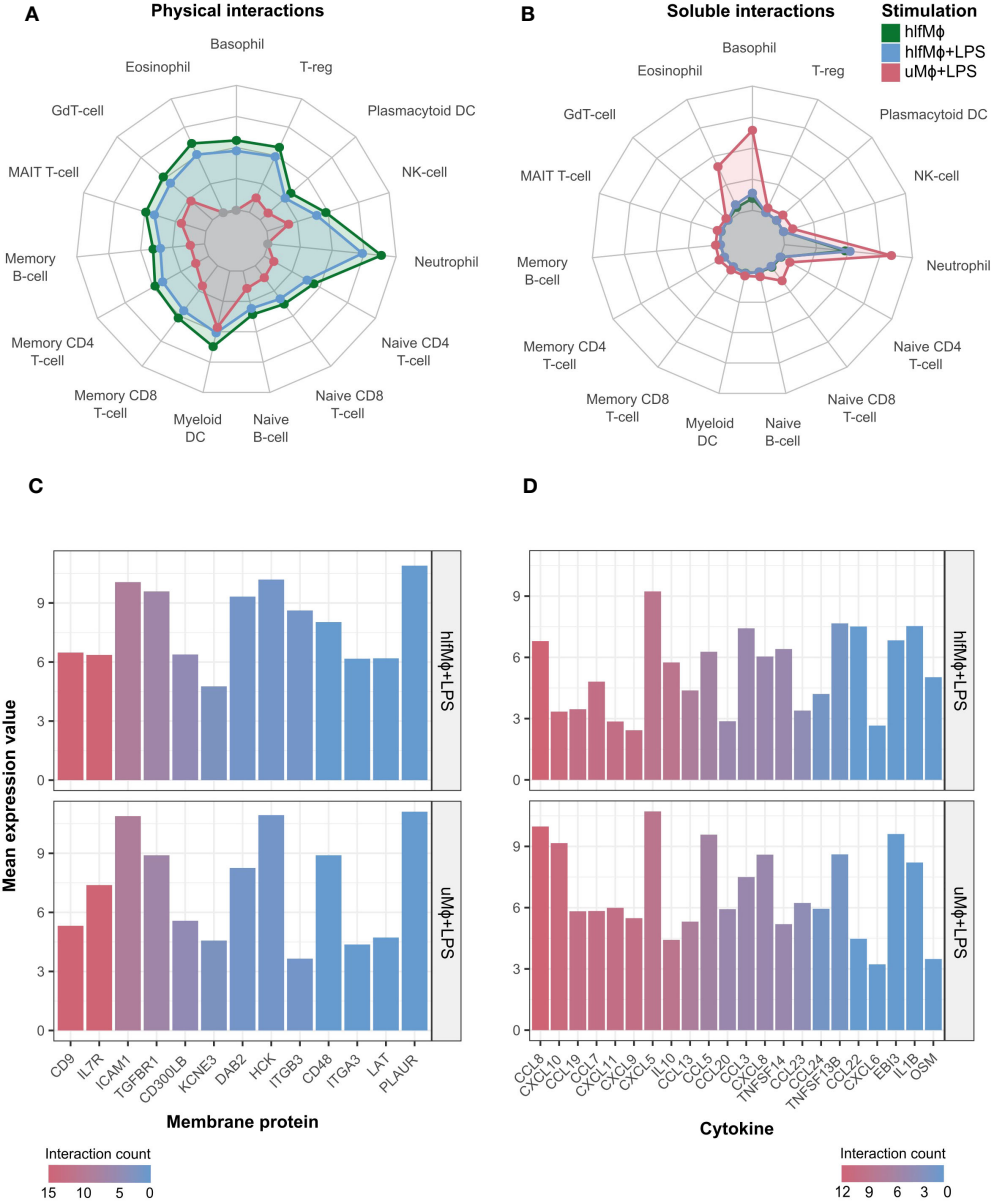
Propensity for soluble and physical cell-cell interaction is markedly altered upon hLF-treatment. Cell-cell interaction probability was manually computed using data deriving the publicly available databases ImmPort, Human Protein Atlas, ImmuneDB, and KEGG. Radar charts depicting cell-cell interaction probability in arbitrary values between differentially treated macrophages in comparison to uMϕ were generated for physical **(A)** and soluble **(B)** cell-cell interactions. Moreover, bar charts visualizing the mean expression values of the most important and differentially regulated interaction molecules between hlfMϕ + LPS and uMϕ + LPS contributing to physical **(C)** or soluble **(D)** cellular crosstalk were created. Bars are ordered and color-coded according to the number of cells respective genes mediate interactions with, denoted as “interaction count”.

hlfMϕ displayed a markedly increased probability for physical cell-cell interactions when compared to uMϕ and uMϕ+LPS. These physical interactions were biased towards granulocytes, regulatory T-cells, and myeloid dendritic cells (**Figure 5A**) – although to a markedly lesser extent than soluble interactions (**Figure 5B**). Indeed, treatment with hLF strongly increased the capability for soluble interaction with neutrophilic granulocytes when compared to uMϕ, arguing for extensive crosstalk between these cell types upon hLF exposure. Moreover, large differences between uMϕ+LPS and hlfMϕ+LPS were observable for both physical and soluble interaction propensities. Detailed analysis of interactome differences between these two conditions revealed differential expression of *CD9*, *IL7R*, *ICAM1*, *TGFBR1*, and *CD300LB* on macrophages as drivers of hLF-induced alterations in physical cell-cell interactions (**Figure 5C**). Regarding cytokines, downregulation of chemoattractants *CCL8*, *CXCL10*, *CCL19*, *CCL7*, and *CXCL9* – amongst others – mediated the decreased probability for soluble interaction observable in hlfMϕ+LPS, when compared to uMϕ+LPS (**Figure 5D**).

Highlighting our previous findings, upregulation of the propensity for physical cell-cell interaction and concomitant downregulation of LPS-mediated soluble interactions argue for hLF-induced polarization of neonatal macrophages into a tissue-homeostatic phenotype.

## 4 Discussion

In our experimental model, differentiation of macrophages in the presence of hLF induced a distinct transcriptional program capable of repressing changes in gene expression provoked by LPS stimulation. HLF’s functional signature involved changes in cell cycle regulation, induction of IL-4/IL-13-like signaling, and modified expression of molecules known to mediate extracellular matrix interactions, efferocytosis, and inflammation. Interestingly, in terms of global immune function and cellular interactome, hLF exhibited a ‘Janus-faced’ character. This duality was characterized by the induction of a basal interaction-prone phenotype incapable of mounting adequate immune synergisms upon TLR4-engagement by LPS. Congruent with previously published data by our group, these findings argue for the polarization of monocyte-derived macrophages into a quiescent state upon treatment with hLF. The results underline the physiological and trajectorial complexity of human lactoferrin as a soluble modulator of macrophage function at the peripheral site of infection and a promotor of immune-homeostasis and tissue integrity within the intestine upon birth.

It Is well established that commensal colonization of the neonatal gut poses an immunological challenge to a hitherto naïve immune system. Thus, it is very likely that physiological mechanisms exist that prevent the development of excessive inflammation, disruption of intestinal barrier function, and onset of diseases such as necrotizing enterocolitis (NEC). Macrophages play a key role in TLR4-mediated induction of intestinal inflammation upon challenge with pathogen-associated molecular patterns (PAMPs) such as LPS (37). In this regard, their polarization towards pro-inflammatory M1- and tissue-homeostatic M2-phenotypes has been shown to be associated with the development of NEC, and modulation proposed as a possible treatment strategy for excessive gut-inflammation (38, 39). As blood monocytes that migrate into the developing gut (40, 41) encounter abundant hLF from colostrum, we aimed to investigate whether hLF modulates the function of these newly differentiating macrophages to prevent excessive gut inflammation upon LPS challenge, the main PAMP associated with gram-negative bacteria.

hlfMϕ displayed cell cycle arrest in the G2/M phase – a condition recently shown to promote an M2-like phenotype and tissue-homeostatic functions (36). Interestingly, this state has been associated with enhanced expression of *FN1*, a molecule also markedly upregulated in our hlfMϕ (36). This finding aligns well with the downregulation of gene-regulatory proteins *BRCA2* and *BARD1*, molecules that promote G1- and S-phase progression (42, 43), and *ASH2L*, an inductor of G2-phase exit (44). From a functional point of view, enrichment of IL-4 and IL-13 signaling, enhanced expression of *SOCS3*, a repressor of proinflammatory cytokine secretion (45), and increase in *JAK3* production, also argue for polarization of cells towards a tissue-homeostatic M2 phenotype. Likewise, we also observed the upregulation of membrane proteins *SIGLEC7*, *SDC4*, *S100A9*, and *TNFRSF9*, molecules associated with the development of an M2-phenotype (46–50).

Complementing its gut-protective role, macrophages differentiated in the presence of hLF displayed enhanced expression of IL-10, an interleukin robustly associated with gut epithelial barrier restoration, suppressor of intestinal inflammation, and mitigator of NEC development (51, 52). Moreover, hlfMϕ likely induces Th2- immunity via expression of *CCL22*, a molecule shown to promote chemotaxis of CCR4-positive Th2-skewed lymphocytes (53), and *TNFSF15*, a cytokine relevant to the expansion of innate lymphoid cells type 2 (ILC2) (54). In the context of gut immunity, Th2-skewed lymphocytes are known to produce cytokines involved in the maintenance of gut-epithelial barrier homeostasis and mitigation of inflammation (55, 56). Interestingly, *TNFSF15* has also been shown to trigger the release of IL-22 by ILC3 (57). As reduction of IL-22 producing RORγt^+^NKp46^+^ ILC3 has recently been linked to the development of NEC, hLF-induced *TNFSF15* secretion in macrophages might convey an important gut-protective role (58).

Underlining hLF’s ‘Janus-faced’ character, microarray analysis also revealed pro-phagocytic signatures via upregulation of *PLAUR*, *LAT*, and downregulation of *CD180* (59–61). While in the context of gut homeostasis this function may aid the clearance of apoptotic cells and mitigate inflammation, upregulation of these molecules might also reflect hLF’s function as a soluble mediator in the periphery. This theory is also supported by hLF-induced priming of macrophages for cytokine-induced immune interactions through enhanced *IL1R1* expression and elevated transcription of pro-inflammatory molecules *OSM*, *IL-1B*, *CCL3*, *CCL7*, and *CXCL5* (62–65).

Although contradictory at first glance, hLF-induced priming of macrophages for pro-inflammatory activity and concomitant global transition towards a tissue homeostatic phenotype seems physiologically reasonable. Considering the high amount of hLF synthesized by granulocytes at the site of infection (5), hLF-induced upregulation of transcripts relevant to phagocytosis, proinflammatory cytokine signaling, and cell migration may aid pathogen clearance. Consequently, as inflammation and tissue damage progress, a more global transcriptional program drives macrophages into a state of immunological anergy and homeostatic function – as represented by a slow, cell-cycle specific transcriptional transition towards an M2 phenotype (36). The observed transcriptional response is also in line with evidence linking hLF-function to basal TLR-4 activation via its carbohydrate chains and suppression of TLR4 signaling upon endotoxemia through its peptide moiety (66). To specifically delineate the molecular mechanisms that govern LPS irresponsiveness upon hLF exposure in human macrophages, future studies would benefit from investigating the persistence of immunological effects upon withdrawal of hLF from culture medium and incubation with different hLF-moieties.

**Figure 6:**
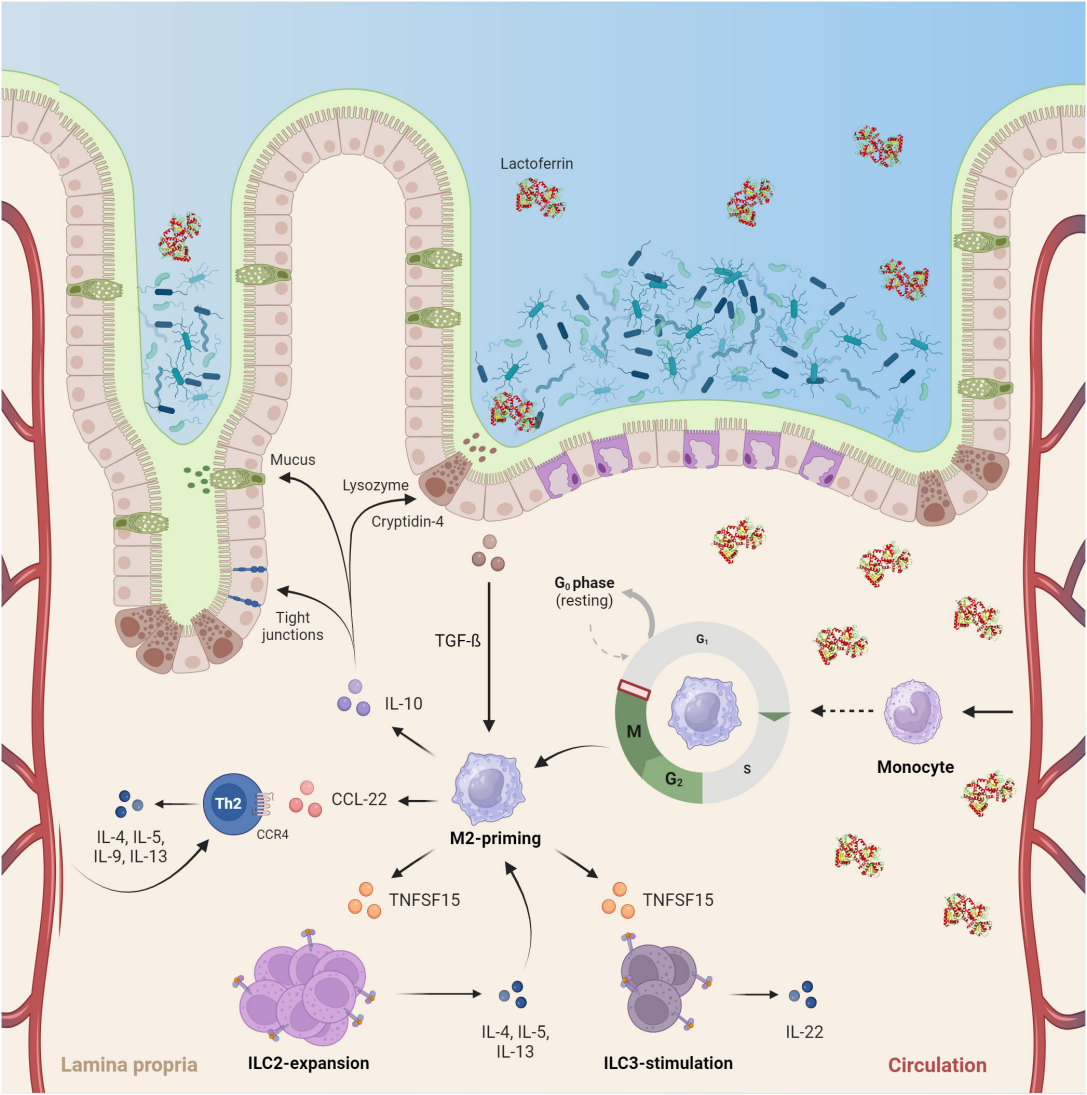
HLF exerts a pivotal role in the developing neonatal intestine by inducing immune-anergy and promoting the development of an anti-inflammatory tissue microenvironment. Upon birth, commensal colonialization of the intestine and confrontation of a hitherto naïve immune system with bacterial antigens occurs. High concentrations of lactoferrin encountered in colostrum (up to 7 g/L) not only directly alter bacterial colonization via iron-sequestration within the gut lumen, but also induce a quiescent M2-like phenotype in human macrophages. This function is driven by cell-cycle arrest in the G2/M-phase and enterocyte-dependent TGF-beta secretion. Subsequently, neonatal gut macrophages adapt a quiescent tissue-homeostatic phenotype and promote Th2-skewed immunity by inducing ILC2 expansion and Th2 recruitment. Moreover, they stimulate ILC3 to produce IL22, a mechanism proposed to counteract the development of necrotizing enterocolitis. Consequently, the cytokine microenvironment created by these cells ultimately aids mucosal barrier function via induction of tight-junctions, mucus production and lysozyme secretion – thus, protecting neonates from excessive inflammation upon commensal colonialization of the intestine (Created with BioRender.com).

The observed dual function of hLF is also consistent with its ontogenetic aspect. Given the high abundance of hLF in colostrum, breastfeeding and uptake of this nutrient lead to uniquely elevated concentrations within the gut. This primes intestinal macrophages towards tolerance, induces the migration and proliferation of ILCs and Th2-skewed lymphocytes, and prohibits the instantaneous development of gut inflammation. The near-total abrogation of LPS-induced changes on the transcriptome of hlfMϕ encountered in all experiments also supports this notion. Thus, based on our data and available literature we propose a mechanistic framework of homeostatic hlfMϕ-function within the neonatal gut (**Figure 6**).

As for study limitations, although the data have been quality controlled ahead of analyses to account for unspecific signals (16), the sample size (n = 4) does not allow correction for donor-specific effects. The potential influence of donor-variation on experimental results is also depicted in **Figure 1E**, where some data-points lie outside the respective confidence intervals. Future studies would benefit from including a larger number of biological replicates to allow for even more stringent exclusion of experimental outliers and determination of potential donor-specific effects. Additionally, while our analysis shows a clear abrogation of TLR4 signaling upon differentiation of macrophages in the presence of hLF, the experimental setup does not consider stimulation of other innate signaling pathways or synergistic signaling effects elicited by whole bacteria. Therefore, even though E.coli has been shown to be a highly abundant strain during early colonialization upon vaginal delivery (67) and LPS arguably serves as a model agonist for TLR engagement by this bacteria (68), future studies would benefit from also investigating transcriptional responses after stimulation with gram-positive bacteria such as Staphylococci and Enterococci to provide a more holistic immunological framework for hLF-function.

Nevertheless, our study provides compelling evidence for the role of hLF as a soluble modulator of macrophage activity. The substantive transcriptional effects hLF exerted on neonatal macrophages, including the near-complete abrogation of pro-inflammatory responses to LPS-treatment, likely reflect the molecule’s ontogenetic importance as an immunosuppressive agent within the developing neonatal gut. Thus, exposure of infants to high concentrations of hLF via uptake of colostrum may enable safe intestinal bacterial colonialization and prevent the development of inflammation and disease. Noteworthy, the considerable effects observed in our study not only confer relevance to hLF but also underline the importance of macrophage function in early-life development - marking this immune-subset a promising therapeutic target in the prevention of inflammation and autoimmunity in infants.

## Data availability statement

The datasets presented in this study are readily available from ArrayExpress under the accession number E-MTAB-13175. The R-script and respective input data files are available from https://github.com/wisgrill-lab/Transcriptomic-analysis-identifies-lactoferrin-induced-quiescent-circuits-in-neonatal-macrophages.

## Ethics statement

The studies involving humans were approved by Ethics committee of the Medical University of Vienna (ref. 1923/2012). The studies were conducted in accordance with the local legislation and institutional requirements. The participants provided their written informed consent to participate in this study.

## Author contributions

ME: Data curation, Formal Analysis, Methodology, Software, Visualization, Writing – original draft, Writing – review & editing. IW: Conceptualization, Investigation, Methodology, Writing – review & editing. MD: Conceptualization, Investigation, Writing – review & editing. EF: Writing – review & editing. AF: Resources, Writing – review & editing. HK: Resources, Writing – review & editing. AB: Conceptualization, Project administration, Resources, Supervision, Writing – review & editing. LW: Conceptualization, Investigation, Methodology, Project administration, Resources, Software, Supervision, Visualization, Writing – original draft, Writing – review & editing.
