# Supplementary figures and images for: Transcriptomic analysis identifies lactoferrin-induced quiescent circuits in neonatal macrophages
Source: Front Immunol. 2023 Oct 6;14:1276173. doi: 10.3389/fimmu.2023.1276173 (PMC10590118; doi:10.3389/fimmu.2023.1276173)

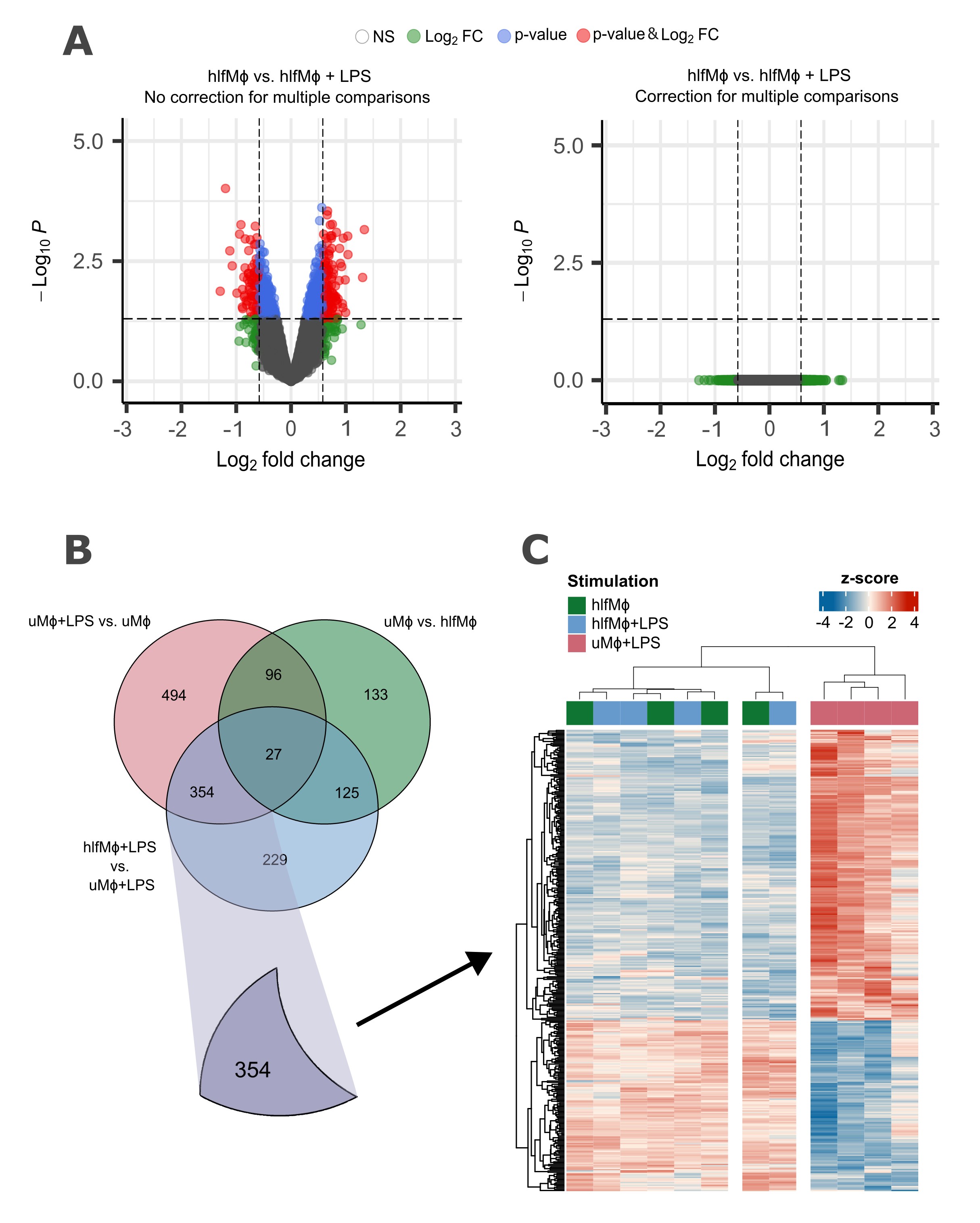

Supplement: Supplementary file 1 [file Image_1.jpeg]
